# Micro-Structure and Thermomechanical Properties of Crosslinked Epoxy Composite Modified by Nano-SiO_2_: A Molecular Dynamics Simulation

**DOI:** 10.3390/polym10070801

**Published:** 2018-07-20

**Authors:** Qing Xie, Kexin Fu, Shaodong Liang, Bowen Liu, Lu Lu, Xueming Yang, Zhengyong Huang, Fangcheng Lü

**Affiliations:** 1 State Key Laboratory of Alternate Electrical Power System with Renewable Energy Sources, North China Electric Power University, Baoding 071000, China; fkx_ncepu@foxmail.com (K.F.); liangshao9564@163.com (S.L.); bowensmz@hotmail.com (B.L.); andrewlu149@163.com (L.L.); 13582251628@126.com (F.L.); 2 Department of Power Engineering, North China Electric Power University, Baoding 071003, China; yxmhhx@163.com; 3 State Key Laboratory of Power Transmission Equipment & System Security and New Technology, Chongqing University, Chongqing 400044, China; huangzhengyong@cqu.edu.cn

**Keywords:** epoxy resin composites, nano-SiO_2_, molecular dynamics, crosslinked structure, free volume, segment motion, glass transition temperature, coefficient of thermal expansion, mechanical properties

## Abstract

Establishing the relationship among the composition, structure and property of the associated materials at the molecular level is of great significance to the rational design of high-performance electrical insulating Epoxy Resin (EP) and its composites. In this paper, the molecular models of pure Diglycidyl Ether of Bisphenol A resin/Methyltetrahydrophthalic Anhydride (DGEBA/MTHPA) and their nanocomposites containing nano-SiO_2_ with different particle sizes were constructed. The effects of nano-SiO_2_ dopants and the crosslinked structure on the micro-structure and thermomechanical properties were investigated using molecular dynamics simulations. The results show that the increase of crosslinking density enhances the thermal and mechanical properties of pure EP and EP nanocomposites. In addition, doping nano-SiO_2_ particles into EP can effectively improve the properties, as well, and the effectiveness is closely related to the particle size of nano-SiO_2_. Moreover, the results indicate that the glass transition temperature (*T_g_*) value increases with the decreasing particle size. Compared with pure EP, the *T_g_* value of the 6.5 Å composite model increases by 6.68%. On the contrary, the variation of the Coefficient of Thermal Expansion (CTE) in the glassy state demonstrates the opposite trend compared with *T_g_*. The CTE of the 10 Å composite model is the lowest, which is 7.70% less than that of pure EP. The mechanical properties first increase and then decrease with the decreasing particle size. Both the Young’s modulus and shear modulus reach the maximum value at 7.6 Å, with noticeable increases by 12.60% and 8.72%, respectively compared to the pure EP. In addition, the thermal and mechanical properties are closely related to the Fraction of Free Volume (*FFV*) and Mean Squared Displacement (*MSD*). The crosslinking process and the nano-SiO_2_ doping reduce the *FFV* and *MSD* value in the model, resulting in better thermal and mechanical properties.

## 1. Introduction

With the growing expansion of electrical equipment applications, the disadvantages of the Epoxy Resin (EP) insulating materials such as Diglycidyl Ether of Bisphenol A resin/Methyltetrahydrophthalic Anhydride (DGEBA/MTHPA) have become increasingly obvious. Some of the properties, including mechanical and thermal properties, are often not able to meet the ever-increasing requirements of electrical equipment [1,2,3,4]; while, the emergence of nanomaterials and the development of nanotechnology in recent years have provided new ways to improve the comprehensive properties of EP [5,6]. Compared with traditional materials, the distinguishing structure of nanomaterial has provided a set of unique features, such as the small size effect, quantum size effect and surface effect [7,8], which further enhance the performance in various aspects of physics and chemistry. The nanocomposites obtained by combining the nanofillers with the polymer possess the advantages of both the nanomaterial and the polymer itself. Numerous scholars have carried out experimental studies on the improvement of EP thermal properties, mechanical properties, electrical properties, etc. [9,10]. The studies emphasized the effects of filler type, particle size, shape and dosage on the properties of the composite EP [11,12]. Despite the progress made by the above researchers, there is no unified understanding of the interface mechanism of EP nanocomposites. Therefore, it is significant to further conduct research on the relationship between the structure and properties of EP nanocomposite, as well as investigating the underlying mechanism of action.

In recent years, molecular simulation technologies have continued to progress and develop, which, at the molecular level, possess obvious advantages in the study of the microstructure and macro-properties of composite polymer materials [13,14,15]. In particularly, the Molecular Dynamics (MD) method has been applied to the study of EP by various scholars [16,17,18,19,20]. In terms of EP composites, some scholars have studied the glass transition and thermoelasticity of SiC/EP nanocomposites by molecular simulation. It is found that the glass transition temperature (*T_g_*) and the Coefficient of Thermal Expansion (CTE) of the composites is lower than that of the pure EP [21]. Chen Shenghui et al. [22] have constructed an uncrosslinked DGEBA and Isophorone Diamine (DGEBA/IPD) epoxy-carbon nanofiber composite interface model, analyzed the distribution, structure and movement characteristics of the monomer molecules and found that carbon nanofibers can significantly alter the concentration and properties of the EP and curing agent during the interfacial phase. Zhang et al. [20] investigated the influence of silane coupling agent on the structure and thermomechanical properties of the nanocomposites through MD simulation and found that incorporating silica nanoparticles into the EP matrix can significantly improve the mechanical and thermal properties of the composites. Furthermore, the thermomechanical properties were further enhanced by silane coupling agent modification on the surface of fillers. R. Zhu et al. [23] carried out MD research on EPON-862 EP (Bisphenol F epoxy resin) reinforced by the carbon nanotube. It was found that the carbon nanotubes can enhance the mechanical properties of the EP. Moreover, with the increase of the aspect ratio, the enhancement effect is more significant. In summary, MD has become an essential instrument in terms of analyzing the performance of composite materials, which is also expected to provide new approaches for the design and preparation of EP composites with enhanced performance.

SiO_2_ is one of the most common fillers for the EP insulating materials. The EP composite filled with nano-SiO_2_ has improved mechanical and thermal properties [7,24,25]. Although scholars have studied the properties of SiO_2_/EP nanocomposites through experiments [26,27,28,29,30], few involved MD simulations.

In this paper, MD simulations were performed on DGEBA/MTHPA EP filled with nano-SiO_2_. The SiO_2_ fillers were spherical, with particle radiuses of 6.5 Å, 7.6 Å, 8.8 Å and 10 Å, respectively. Based on the MD simulations, the parameters of the models, including the Fraction of Free Volume (*FFV*), Mean Square Displacement (*MSD*), *T_g_*, CTE and mechanical properties, were analyzed. The effects of crosslinking density and nano-SiO_2_ particle size on the microstructure, thermal and mechanical properties of EP were investigated. In addition, the relationship between the structure and properties of composite EP was analyzed. Furthermore, this paper provided theoretical guidance for the design and preparation of EP nanocomposites with enhanced performance.

## 2. Model Construction and Simulation Details

In this section, the simulation steps are synthesized as the following three parts: (1) mix DGEBA and MTHPA according to the actual proportion, and fill the models with nano-SiO_2_; (2) construct the amorphous models of the crosslinked EP/SiO_2_ nanocomposites based on the practical crosslinking reaction mechanism; (3) perform MD simulations of DGEBA/MTHPA/SiO_2_ crosslinked models with different crosslinking densities and filler sizes. The models mentioned in this paper were built using Material Studio (developed by Accelrys).

### 2.1. Reaction Mechanism of EP and Curing Agent

**Figure 1:**
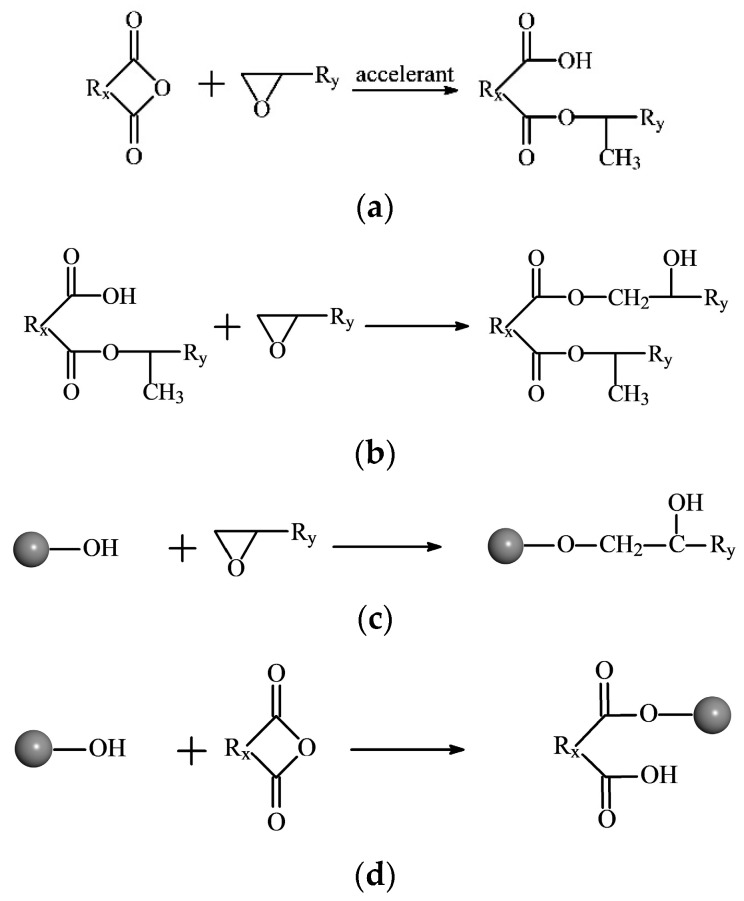
Mechanism of crosslinking reaction between the epoxy, acid anhydride curing agent and nano-SiO_2_. (**a**) Under the effect of trace moisture existing in the system, part of the epoxy groups open and anhydrides hydrolyze to further react with each other and produce monoester containing carboxyl; (**b**) carboxyl reacts with the epoxy groups and generates hydroxyl groups; (**c**) the hydroxyl groups on the surface of SiO_2_ and the generated hydroxyl react with the epoxy groups to generate hydroxyl groups; (**d**) the hydroxyl groups on the surface of SiO_2_ and the generated hydroxyl react with anhydrides to generate carboxylic acids.

Due to the existence of the numerous hydroxyl groups on the surface of nano-SiO_2_, nanofillers were also considered during the crosslinking reaction. The reaction mechanism is shown in Figure 1. By repeating the process in Figure 1c,d, the crosslinking reaction was carried out.

Here, the crosslinking reaction between DGEBA, MTHPA and SiO_2_ was realized through programming. To simplify the process, the following assumptions have been made: (1) The crosslinking density is defined as the ratio of the reacted epoxy groups to the initial epoxy groups. (2) To start the crosslinking process, we introduce the partial product of (a) during the model construction stage (i.e., ignoring Reaction (a) in the crosslinking process) as a crosslinking primer (denoted by DGEBA-MTHPA). As a result, the crosslinking density of the initial model is 10%. (3) The activity of each reaction group is the same. (4) The reaction is diffusion controlled. (5) The reactions are synchronized.

### 2.2. Construction of Crosslinked EP/SiO_2_ Composite Models

**Figure 2:**
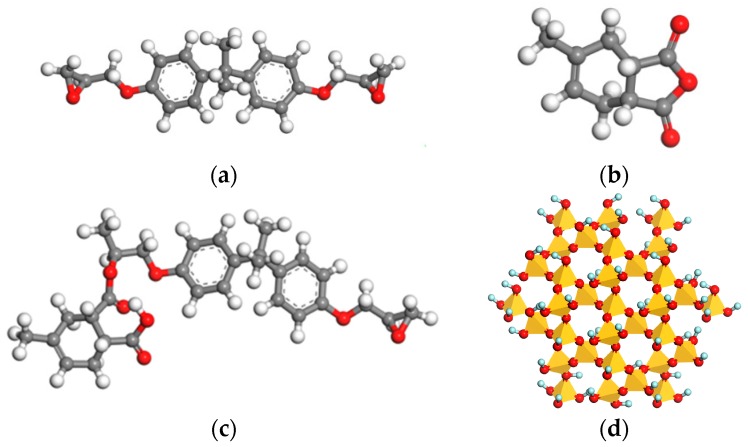
Molecular models. (**a**) Diglycidyl Ether of Bisphenol A resin (DGEBA); (**b**) Methyltetrahydrophthalic Anhydride (MTHPA); (**c**) DGEBA-MTHPA; (**d**) SiO_2_.

Epoxy prepolymer is regarded as a mixture of DGBEA with a polymerization degree of zero and one. In this paper, the polymerization degree of DGEBA was set to zero in the modeling process to simplify the composite model [23]. The modeling process of crosslinked EP/SiO_2_ composite is as follows:

(1) Construct the molecular models of DGEBA, MTHPA, DGEBA-MTHPA and spherical nano-SiO_2_ with radiuses of 6.5 Å, 7.6 Å, 8.8 Å and 10 Å, respectively. Simulate the oxidation reaction of SiO_2_, so that the surface of fillers has massive hydroxyl groups [31,32]. Subsequently, optimize the structure of the above models and obtain the molecular models as shown in Figure 2.
(2) Considering that the actual molar ratio of DGEBA and MTHPA is about 1:2 [20,33], we constructed amorphous molecular models of pure EP and EP nanocomposite with different filler sizes. Using periodic boundary conditions, the initial temperature of the model was set to be 580 K to facilitate the subsequent annealing process. Each nanocomposite model was filled with a nano-SiO_2_ particle to simulate the well-dispersed situation. The mass fraction of SiO_2_ in each system was maintained at 6.5% by adjusting the amount of the ingredients according to Table 1. Further, 10 amorphous molecular models with an initial crosslinking density of 10% were constructed under each system, and their geometric structures were optimized based on the minimum energy principle. The amorphous models with the lowest energy were selected for further simulation and calculation.
(3) Perform a 200-ps MD simulation on each model under the NPT ensemble (the constant temperature and pressure ensemble). The specific details of the MD process are as follows. The time step was 1 fs. The temperature and pressure were controlled at 580 K and 1.0 × 10^−4^ GPa (which is one standard atmospheric pressure) respectively by applying the Andersen and Berendsen methodology [20,34]. In addition, the van der Waals interaction and electrostatic interaction were calculated by the atom-based and Eward method, respectively.
(4) The scriptlet employed for the crosslinking reaction was written according to Section 2.1. The crosslinking was conducted in stages, and the molecular models with different crosslinking densities were obtained by conducting crosslinking reactions multiple times.

### 2.3. MD and Annealing Simulation

Molecular models with crosslinking densities of 10%, 34%, 67% and 96% were further selected for MD processing. First, MD simulations of 200 ps were performed under the NVT ensemble (constant volume and temperature ensemble) and NPT ensemble successively, in order to eliminate the stress generated during the crosslinking process. The time step was 1 fs, and the temperature was 580 K. In addition, the pressure in the NPT ensemble was controlled at 1.0 × 10^−4^ GPa.

**Figure 3:**
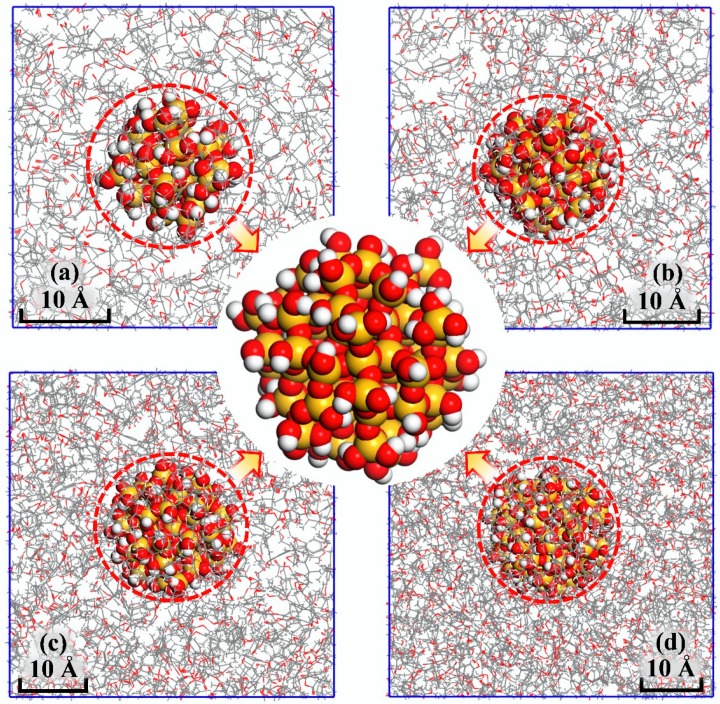
The crosslinked network in Epoxy Resin (EP) nanocomposite models. (**a**) For 6.5 Å; (**b**) for 7.6 Å; (**c**) for 8.8 Å; (**d**) for 10 Å.

Subsequently, anneal simulations were performed on the above models from 580 K–280 K to extract the information of density, volume and temperature for thermal property analysis. The annealing rate was set to be 10 K/100 ps, i.e., an NPT ensemble MD simulation of 100 ps was performed after each temperature decrease by 10 K. The models of EP composite at 300 K are shown in Figure 3.

## 3. Simulation Results and Model Parameters

### 3.1. Free Volume

The free volume theory [35] holds that the volume of a liquid or solid substance, *V_T_*, consists of two parts, i.e., the volume occupied by the molecule denoted as *V_o_*, and the free volume *V_f_*, where:

(1) $$V_{T}=V_{o}+V_{f}$$

*V_f_* is the intermolecular space, which disperses in the form of a hole among the materials and provides space for molecule movements. It also enables the movement of the molecular chain. The influence of nanoparticles on the free volume of the polymer was relatively complex in the following two aspects. First, the nanoparticle occupied a considerable part of the volume of the system and increased the distance between chain segments, which hindered the movement and stacking of molecular segments and reduced the free volume. Moreover, the nanoparticle can participate in the crosslinking reaction of the EP system and fix molecular chain segments, therefore expanding the crosslinked network and increasing the free volume. The above impacts existed in all the EP nanocomposite materials. Therefore, the increase or decrease of the free volume significantly depended on the properties of the nanoparticles.

The volumes of different models differed greatly due to the different numbers of molecules. As a result, the free volumes were different, as well. Therefore, to facilitate the comparison of the free volume characteristics of different systems, the fraction of free volume (*FFV*) was introduced to characterize the relative size of the free volume. The expression is [35]:

(2) $$FFV=\frac{V_{f}}{V_{0}+V_{f}}\times 100\%$$

**Figure 4:**
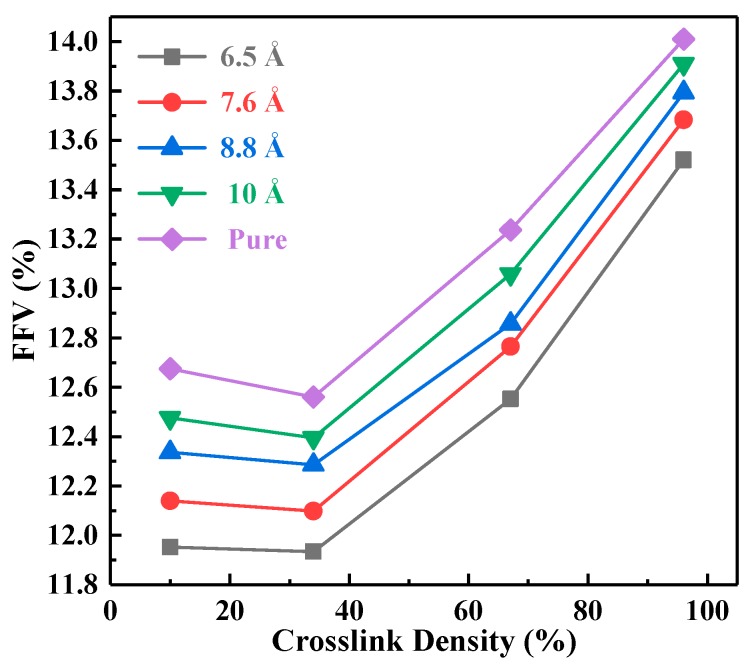
The Fraction of Free Volume (*FFV*) of each system with different crosslinking densities.

Here, the *FFV* of EP/nano-SiO_2_ composites with different particle sizes were calculated at 300 K. The variation trend of the *FFV* with the crosslinking density is shown in Figure 4.

According to Figure 4, with the increase of crosslinking density, the *FFV*s of pure EP and composites decrease first and then increased. The reason for the above trend is as follows. In the initial stage of the crosslinking reaction, the free volume in the crosslinking network was occupied by a dangling chain appearing [36,37]; therefore, the *FFV* of the system decreased slightly. Subsequently, the formation of a crosslinking network and the decrease of the dangling segment led to the increase of the free volume. On the other hand, the *FFV* of the nanocomposites was lower than that of the pure EP. The underlying cause is that the nanoparticles occupied a considerable part of the space in the system, resulting in a smaller range of segmental motion and a decrease in the *FFV*. Furthermore, the *FFV* decreased with the decreasing particle size. This was due to the final crosslinked network being generated by the covalent links between SiO_2_ and EP matrix in the system, and the interaction tended to enhance as the particle size decreased. Previous studies have suggested that the mechanical properties of the polymers depended on the free volume to a certain extent. The smaller the free volume was, the better the performance could be [35,38]. Therefore, it can be inferred that the mechanical properties of the system containing fillers were better than that of the pure EP system. Moreover, the smaller the particle size was, the better the mechanical property was when the crosslinking density was relatively high.

### 3.2. Segment Motion

The molecules of the models were constantly moving throughout the MD process. Some research has proven that there is a correlation between the strength of the segment motion and the mechanical properties of the polymer. Specifically, the enhancement of molecular segment motion may reduce the mechanical modulus of EP [39]. Moreover, the mean squared displacement (*MSD*) is defined as a microscopic parameter to characterize the motion capability of each atom or molecular segment in the system, which is defined as the mean squared displacement of the molecules or segments, respectively. In general, the slope of the *MSD* curve indicates the strength of the polymer molecule segment motion. The *MSD* in a system containing *N* atoms can be described as follows [38]:

(3) $$MSD=\frac{1}{3N}\sum_{i=0}^{N-1}\left[{\left|\left(\vec{R_{i}})(t)-(\vec{R_{i}})(0\right)\right|}^{2}\right]$$

where ${\vec{R}}_{i}\left(t\right)$ and ${\vec{R}}_{i}\left(0\right)$ denote the displacement vector of any atom *i* at time *t* and the initial time in the system, respectively. In this paper, the *MSD* values of systems with different crosslinking densities, particle sizes and temperature in the first 30 ps of MD simulations under the NPT ensemble were investigated.

**Figure 5:**
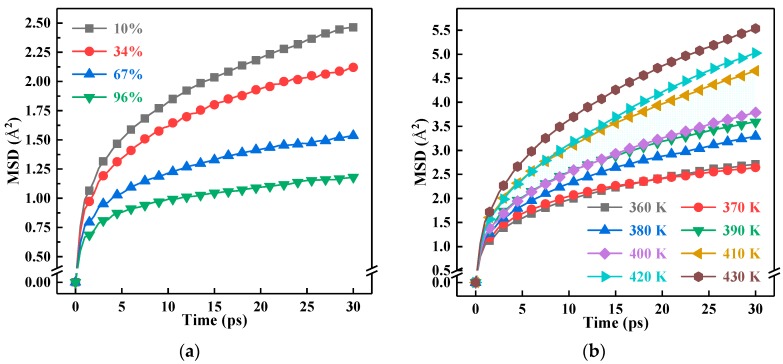

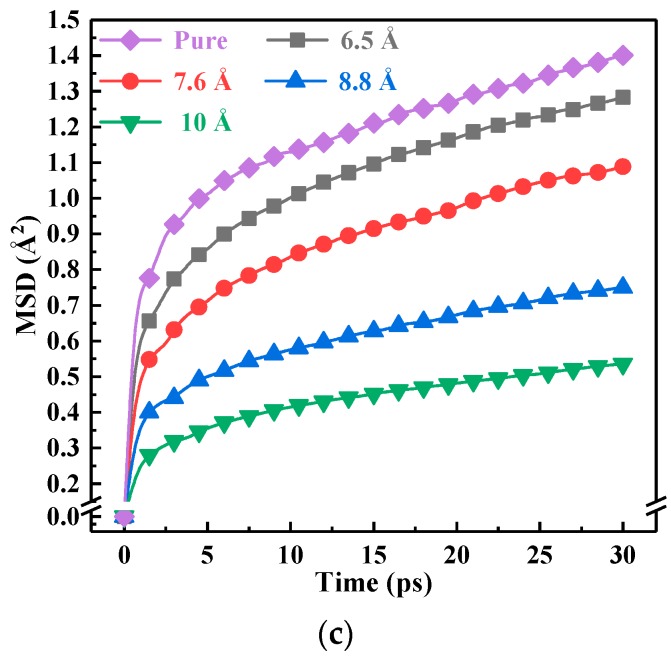
The variation of Mean Squared Displacement (*MSD*) values with time. (**a**) *MSD* values of the 6.5 Å system at 300 K with different crosslinking densities; (**b**) *MSD* values of the 67% crosslinking density system with 6.5 Å particle sizes at different temperatures; (**c**) *MSD* values of the 67% crosslinking density system at 300 K with different particle sizes.

The *MSD* values of the 6.5 Å system under different crosslinking densities are shown in Figure 5a. In addition, the trends of *MSD* and crosslinking density in other systems were similar. As the crosslinking density increased, the *MSD* values of EP and its nanocomposites gradually decreased, which indicated that the crosslinked structure limited the molecular motion in the model. To analyze the effect of particle size on the segment motion, the *MSD* values of different particle size models were normalized based on the number of atoms in the pure EP system, as suggested in Table 1. The processed *MSD* values are shown in Figure 5c. The segment motion was negatively correlated with the particle size. It can be seen that nano-SiO_2_ dopants can significantly limit the movement of the molecular segment in the material, and the restriction will be more pronounced as the particle size increases. In addition, temperature had a significant effect on segmental motion. Particularly, temperature had an apparent effect on the segmental motion of EP and its composites, as well. With the increasing of temperature, *MSD* values increased continuously, as shown in Figure 5b. This tendency was due to the thermal motion of the molecules. As the temperature increased, the thermal motion of the molecule increased, which caused the increasing of *MSD*. Furthermore, in the temperature range of 400 K–410 K, the variation of *MSD* values was the most conspicuous, which was related to the glass transition of the materials.

### 3.3. Glass Transition Temperature

The glass transition temperature (*T_g_*) refers to the temperature for the transition from the glassy state to the rubbery state of the amorphous polymer or vice versa, which is a critical thermal performance index of EP materials. When EP transitions from the glassy state to the rubbery state at temperature *T_g_*, its performance completely changes due to the state conversion. Therefore, obtaining the accurate value of *T_g_* is an important prerequisite for thermal performance comparisons across different systems. In this paper, the density-temperature linear fitting and *MSD* curve method were used to accurately predict the *T_g_* of each system.

**Figure 6:**
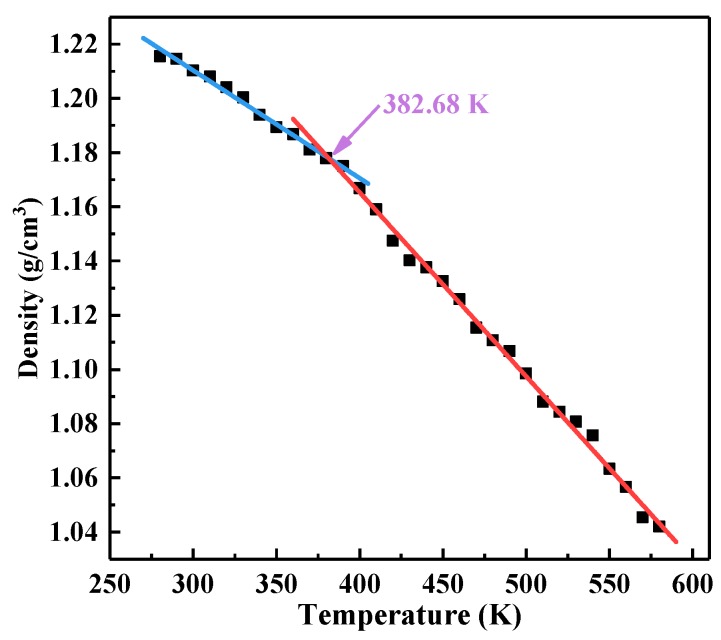
Linear fitting of the density-temperature curve in each system.

1. Density-temperature linear fitting method:

As the temperature increased, the density of the EP showed a linear decrease. However, the density decrease rates in the glassy and rubbery state were different [40,41]. We plotted the density-temperature scatter diagram based on the temperature and density extracted during the annealing process. Figure 6 presents the density-temperature curve of the 67% crosslinked pure EP, where there is an obvious inflection point in each scatter plot. The scatter points on the two sides of the inflection point are linearly fitted, and the turning point of the two lines is the *T_g_* value of the material.

2. *MSD* curve method:

Studies have shown that as the temperature increases, the *MSD* gradually increases. When transitioning from the glassy state to the rubbery state, the motion state of the molecules in the system will change drastically. This mutation reflecting on the *MSD* curve was a sudden jump of the *MSD* values at the temperature interval above and lower than *T_g_*. Therefore, by investigating the *MSD* characteristics of the polymers at different temperatures and observing the temperature range of these curves, the temperature range of *T_g_* can be predicted [42].

The *MSD* of the pure EP and EP nanocomposite systems at various temperatures was calculated at 67% crosslinking density. To stimulate the process, the temperature interval in this paper was set to be 10 K. As shown in Figure 5b, taking the 6.5 Å EP composite system as an example, there was a wider gap between the 400 K and 410 K *MSD* curves. Therefore, we concluded that the *T_g_* value of the 6.5 Å EP composite system was in the (400 K, 410 K) interval.

*T_g_* values obtained by the density-temperature linear fitting method and the *T_g_* intervals obtained by the *MSD* curve method are summarized in Table 2. The results of the two methods were highly consistent with each other. In addition, the *T_g_* value of the pure EP with 67% crosslinking density was 382.68 K, which was highly close to the actual *T_g_* value of DGEBA/MTHPA epoxy [43]. It can be assumed that 67% was close to the industrial crosslinking density of the pure DGEBA/MTHPA system.

**Figure 7:**
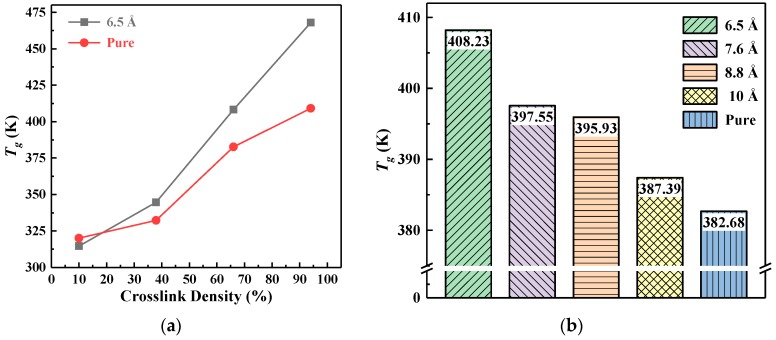
The *T_g_* value of EP and its composites. (**a**) The *T_g_* value of pure EP and EP composite with 6.5 Å nano-SiO_2_ at different crosslinking densities; (**b**) the *T_g_* value of pure EP and EP composite with different particle sizes at 67% crosslinking density.

The *T_g_* values of EP and its composites for different particle sizes and crosslinking densities are summarized in Figure 7. According to Figure 7a, the *T_g_* value of pure EP and EP composite with 6.5 Å nano-SiO_2_ increased as the crosslinking reaction went on. Moreover, when the crosslinking density was greater than 34%, the increase rate of the *T_g_* value with crosslinking density was higher than that in the initial stage of the crosslinking process. Therefore, increasing the crosslinking density can significantly improve the *T_g_* value of EP and its composites. According to Figure 5a, the segmental motion in the model was limited by the crosslinked structure, and the flexibility of segments decreased. Therefore, it is difficult to alter the conformation of the models. The higher the crosslinking density, the stronger the effect was and, ultimately, the higher the *T_g_* value.

Figure 7b shows the *T_g_* value of pure EP and EP composite with different particle sizes at 67% crosslinking density. Nano-SiO_2_ can significantly improve the *T_g_* value of EP composites. Particularly, the *T_g_* value of composites doped with 6.5 Å SiO_2_ increased by 6.68%. Analysis suggested that the excellent thermal properties of nano-SiO_2_ can increase the *T_g_* value of the composites. On the other hand, the inorganic-organic interface integration between the filler and the EP matrix was an important factor to increase the *T_g_* value of the material. With the decrease of the particle size, the small size effect of nanoparticles was more intense, and the interface effect was strengthened. Therefore, the interfacial bonding between the nano-SiO_2_ and the matrix was stronger, resulting in higher *T_g_* values.

### 3.4. Coefficient of Thermal Expansion

Coefficient of Thermal Expansion (CTE) refers to the ratio of the increment in the length of the unit temperature and its length at 300 K, which is a critical index factor to measure the thermal stability of materials. The calculation formula is as follows [21]:

(4) $$\mathrm{CTE}=\frac{1}{V_{0}}{\left(\frac{\partial V}{\partial T}\right)}_{P}$$

where *V*_0_ is the volume at 300 K and *P* is taken as the standard atmospheric pressure. The relationship between the volume and temperature of different EP systems was linearly fitted to obtain the values of ∂V/∂T.

**Figure 8:**
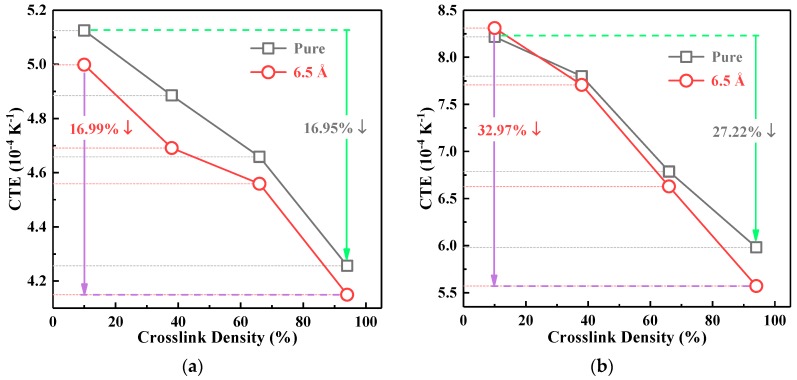
The Coefficient of Thermal Expansion (CTE) in the glassy state and rubbery state. (**a**) Glassy state; (**b**) rubbery state.

Taking pure EP and the 6.5 Å composite as examples, the CTE at the glassy state and rubbery state obtained by Equation (4) are shown in Figure 8. According to Figure 8, the CTE of the two states decreased significantly with the increase of the crosslinking density. Specifically, when the crosslinking density increased from 10%–96%, the CTE of pure EP and the 6.5 Å composites in the glassy state decreased by 16.95% and 16.99%, respectively. Correspondingly, those in the rubber state decreased by 27.22% and 32.97%. This was attributed to the 3D network structure formed in the models during the crosslinking reaction, resulting in a reduced flexibility of the molecular chain and bound motion of polymer segments. However, the CTE of EP composites was lower than that of pure EP because of the extremely low CTE of the SiO_2_ crystal itself. In addition, the CTE in the glassy state was smaller than that in the rubbery state due to the different states of free volume, which is consistent with [20,21,32]. In the glassy state, the free volume of the materials was in the frozen state, and the expansion of the occupied volume was the only dominant factor affecting CTE; while, in the rubbery state, both the free volume and the occupied volume contributed to the thermal expansion, which led to the higher CTE in the glassy state.

**Figure 9:**
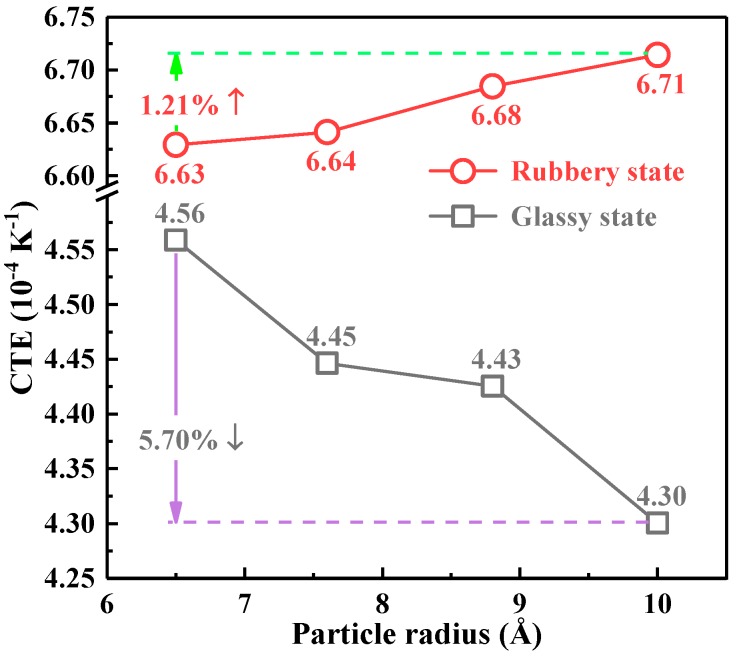
The CTE with different particle sizes under 67% crosslinking density.

On the other hand, the CTE in the glassy and rubbery states showed different trends with the variation of the particle size. As shown in Figure 9, with the increase of the particle size of nano-SiO_2_, the CTE in the glassy state decreased gradually. In the glassy state, the CTE of the 10 Å model was reduced by 5.70% compared to that of the 6.5 Å system and 7.70% compared to that of the pure EP; while the CTE in the rubbery state increased with the particle size. In general, the CTE in the glassy state provided a better referential value considering the fact that the actual working state of the EP materials was the glassy state.

### 3.5. Elastic Moduli

The static constant strain method [44] was used to analyze the mechanical properties of the EP systems in this paper. A slight strain was applied to the system, which was originally in the state of mechanical equilibrium, to enable the model to generate the uniaxial tension and compression deformation along the x-, y- and z-axis, respectively, i.e., shear deformation in the xy-, xz- and yz-plane, correspondingly. The stress-strain relationship obeys Hooke’s law:

(5) σ=Cε

where ***σ*** is the stress vector, ***ε*** is strain vector and *C* is the stiffness matrix. The EP models in the simulation can be regarded as an isotropic material; thus, *C* can be simplified as follows:

(6) $$\left[\begin{array}{cccccc} \lambda +2\mu & 0 & 0 & 0 & 0 & 0\\ 0 & \lambda +2\mu & 0 & 0 & 0 & 0\\ 0 & 0 & \lambda +2\mu & 0 & 0 & 0\\ 0 & 0 & 0 & \mu & 0 & 0\\ 0 & 0 & 0 & 0 & \mu & 0\\ 0 & 0 & 0 & 0 & 0 & \mu \end{array}\right]$$

where *λ* and *μ* are elastic constants and can be obtained from the stiffness matrix. The parameters such as Young’s modulus *E* and shear modulus *G* of an EP system can be obtained from *λ* and *μ*:

(7) $$E=\mu \frac{3\lambda +2\mu }{\lambda +\mu }$$

(8) G=μ

**Figure 10:**
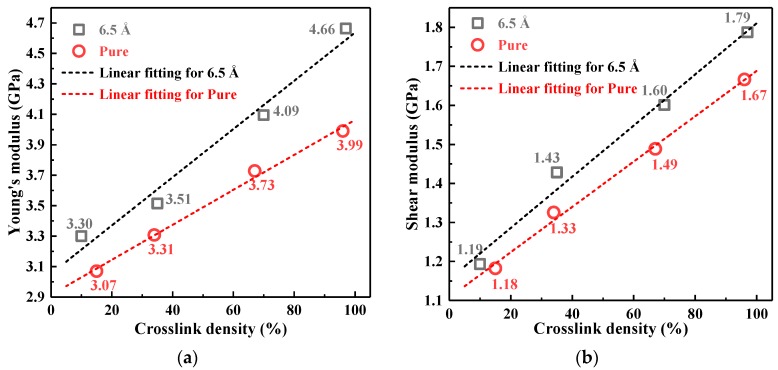
The static mechanical properties of the pure EP and 6.5 Å composites with different crosslinking densities. (**a**) Young’s modulus; (**b**) shear modulus.

The static mechanical properties of the pure EP and composites were investigated at 300 K. Figure 10 shows Young’s modulus and the shear modulus of pure EP and 6.5 Å composites. With the increase of the crosslinking density, the two modulus values increased for all systems. The reason is that the stable 3D polymer network structure formed by the curing agent, EP matrix and nano-SiO_2_ further enhanced the stiffness and mechanical properties of the material. Furthermore, according to the results shown in Figure 8, the crosslinked structure lowered *MSD* and limited the molecular motion in the model, which may result in higher moduli, as well [38]. Moreover, the static mechanical properties of the nanocomposite systems were obviously better than that of the pure EP system, and the modulus growth rates with the crosslinking density of the composites were higher than that of pure EP. The main reason is the high module of nanoparticles, strengthening the epoxy matrix [7].

**Figure 11:**
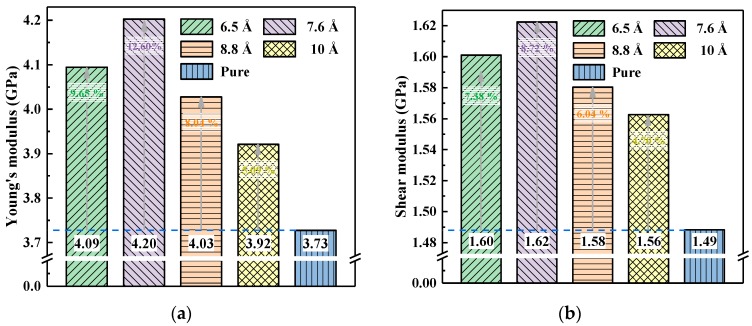
The static mechanical properties of the pure EP and composites with different particle sizes at a crosslinking density of 67%. (**a**) Young’s modulus; (**b**) shear modulus.

The Young’s and shear moduli of the pure EP and composites with different particle sizes at a crosslinking density of 67% are shown in Figure 11. The doping of nano-SiO_2_ with different particle sizes improved the mechanical properties of the DGEBA/MTHPA matrix to varying degrees. Among them, the 7.6 Å SiO_2_ had the best performance, where Young’s modulus and the shear modulus increased by 12.60% and 8.72%, respectively. The phenomena were basically consistent with the results of free volume analysis. The smaller the particle size was, the smaller the free volume was, the higher the moduli could be. However, when the particle size of SiO_2_ was less than 7.6 Å, the Young’s modulus and the shear modulus decreased slightly, which indicated that there were many factors affecting the mechanical properties of the nanocomposites. The interface characteristics produced by different particle sizes may be another factor affecting the mechanical properties, except the free volume.

## 4. Conclusions

In this paper, an automatic crosslinking method for epoxy resin, anhydride curing agent and nano-SiO_2_ was developed, which effectively improved the accuracy of modeling and the molecular simulation efficiency of epoxy composites. The microstructure, thermal and mechanical properties of epoxy resin and its composites were studied by molecular dynamics. The results show that the crosslinking densities of epoxy resin and the doping of nano-SiO_2_ can influence the structure and properties of the materials in many ways. The obtained findings are as follows:

1. The thermal and mechanical properties were improved by increasing the crosslinking density. With the increase of crosslinking density, the glass transition temperature (*T_g_*) increased and the coefficient of thermal expansion (CTE) decreased; in addition, Young’s modulus and the shear modulus of the materials increased, and the mechanical properties were enhanced.
2. Doping nano-SiO_2_ particles into epoxy resin effectively improved the thermal and mechanical properties, and the effectiveness was closely related to the particle size of nano-SiO_2_. The *T_g_* value increased with the decreasing particle size. Compared with pure epoxy resin, the *T_g_* of 6.5 Å composite model was increased by 6.68%. The variation of CTE in the glassy state demonstrated opposite trend as compared with *T_g_* value. The CTE of 10 Å composite model was the lowest, which is 7.70% less than that of pure epoxy. In addition, the mechanical properties first increased and then decreased with the decreasing particle size. Both the Young’s modulus and shear modulus reached the maximum at the 7.6 Å, and increased by 12.60% and 8.72% respectively compared with pure epoxy.
3. The thermal and mechanical properties are closely related to the Fraction of Free Volume (*FFV*) and Mean Squared Displacement (*MSD*). The crosslinking process and the nano-SiO_2_ doping reduced the *FFV* in the model, impeded the deformation and improved the elastic modulus of the system. Moreover, the decreasing *FFV* reduced the *MSD* of the model, limited the segment motion of the molecular chains and made it even harder for the glass transition.

## Figures and Tables

**Table 1:** Initial components of the molecular model of each system.

Particle Size of SiO_2_	Molecular Number of			Atomic Number
(Å)	DGEBA	MTHPA	DGEBA-MTHPA	
none	40	90	10	4650
6.5	40	90	10	4838
7.6	65	146	16	7823
8.8	88	199	23	10,708
10	124	288	40	15,830

**Table 2:** *T_g_* value of 67% crosslink density.

Particle Size (Å)	*T_g_* by Fitting Method (K)	*T_g_* by *MSD* Curve Method (K)
6.5	408.23	400–410
7.6	397.55	390–400
8.8	395.93	390–400
10	387.39	380–390
Pure	382.68	380–390
